# 4-[3-(Isonicotino­yloxy)propoxycarbon­yl]pyridinium diiodidoargentate(I)

**DOI:** 10.1107/S1600536811047295

**Published:** 2011-11-12

**Authors:** Javier Vallejos, Iván Brito, Alejandro Cárdenas, Michael Bolte, Matías López-Rodríguez

**Affiliations:** a Departamento de Química, Facultad de Ciencias Básicas, Universidad de Antofagasta, Casilla 170, Antofagasta, Chile; b Departamento de Física, Facultad de Ciencias Básicas, Universidad de Antofagasta, Casilla 170, Antofagasta, Chile; c Institut für Anorganische Chemie der Goethe-Universität Frankfurt, Max-von-Laue-Strasse 7, D-60438 Frankfurt am Main, Germany; d Instituto de Bio-Orgánica ’Antonio González’, Universidad de La Laguna, Astrofísico Francisco Sánchez N°2, La Laguna, Tenerife, Spain.

## Abstract

The structure of the title compound, (C_15_H_15_N_2_O_4_)[AgI_2_], consists of an organic 4-[3-(isonicotino­yloxy)propoxycarbon­yl]pyridinium cation which has a *gauche*–*gauche* (O/C/C/C—O/C/C/C or GG’) conformation and lies on a twofold rotation axis, which passes through the central C atom of the aliphatic chain, and an inorganic [AgI_2_]^−^ anion. In the complex anion, the Ag^+^ cation is bound to two I^−^ anions in a linear geometry. The anion was modelled assuming disorder around a crystallographic inversion centre near the location of the Ag^+^ cation. The crystal packing is stabilized by a strong inter­molecular N—H⋯N hydrogen bond, which links the cations into zigzag chains with graph-set notation *C*(16) running along the face diagonal of the *ac* plane. The N-bound H atom is disordered over two equally occupied symmetry-equivalent sites, so that the mol­ecule has a pyridinium ring at one end and a pyridine ring at the other.

## Related literature

For a related structure, see: Brito *et al.* (2010 ▶). For conformation definitions, see: Carlucci *et al.* (2002 ▶). For coordination polymers, see: Brito *et al.* (2011 ▶); Albanez *et al.* (2011 ▶). For graph-set notation, see: Bernstein *et al.* (1995 ▶). For polymeric organic-inorganic materials, see: Blake *et al.* (1999 ▶). For mol­ecular geometry calculations, see: Macrae *et al.* (2008 ▶).
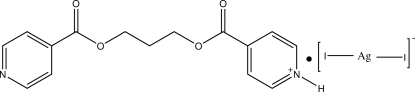


## Experimental

### 

#### Crystal data

(C_15_H_15_N_2_O_4_)[AgI_2_]
*M* *_r_* = 648.96
Monoclinic, 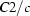

*a* = 14.8788 (7) Å
*b* = 5.4712 (3) Å
*c* = 24.5008 (11) Å
β = 95.347 (4)°
*V* = 1985.81 (17) Å^3^
*Z* = 4
Mo *K*α radiation
μ = 4.14 mm^−1^
*T* = 173 K
0.22 × 0.13 × 0.10 mm

#### Data collection

Stoe IPDS II two-circle diffractometer
Absorption correction: multi-scan (*MULABS*; Spek, 2009 ▶; Blessing, 1995 ▶) *T*_min_ = 0.463, *T*_max_ = 0.682
9000 measured reflections
2158 independent reflections
1821 reflections with *I* > 2σ(*I*)
*R*_int_ = 0.053

#### Refinement

*R*[*F* ^2^ > 2σ(*F* ^2^)] = 0.041
*wR*(*F* ^2^) = 0.119
*S* = 1.03
2158 reflections
124 parameters
H-atom parameters constrained
Δρ_max_ = 1.23 e Å^−3^
Δρ_min_ = −0.76 e Å^−3^

### 

Data collection: *X-AREA* (Stoe & Cie, 2001 ▶); cell refinement: *X-AREA*; data reduction: *X-AREA*; program(s) used to solve structure: *SHELXS97* (Sheldrick, 2008 ▶); program(s) used to refine structure: *SHELXL97* (Sheldrick, 2008 ▶); molecular graphics: *XP* in *SHELXTL* (Sheldrick, 2008 ▶); software used to prepare material for publication: *SHELXL97*.

## Supplementary Material

Crystal structure: contains datablock(s) I, global. DOI: 10.1107/S1600536811047295/zl2424sup1.cif

Structure factors: contains datablock(s) I. DOI: 10.1107/S1600536811047295/zl2424Isup2.hkl

Additional supplementary materials:  crystallographic information; 3D view; checkCIF report

## Figures and Tables

**Table 1:** Hydrogen-bond geometry (Å, °)

*D*—H⋯*A*	*D*—H	H⋯*A*	*D*⋯*A*	*D*—H⋯*A*
N14—H14⋯N14^i^	0.88	1.80	2.684 (7)	176

Symmetry code: (i) 
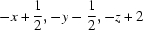
.

**Fig. 1.**
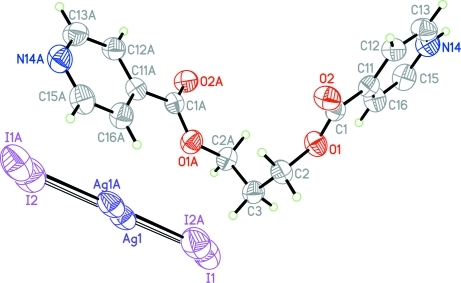
The molecular structure of (I), showing symmetry-complete molecules. Displacement ellipsoids are drawn at the 50% probability level. Symmetry operator for generating equivalent atoms: (A) (-x, y ,3/2 -z ). The two equally occupied components of the disordered AgI2 anion are drawn will full and open bonds.(symmetry code: A: -x,-y+1,-z+1).

**Fig. 2.**
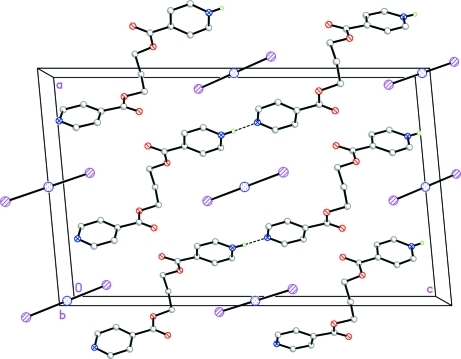
Packing diagram of the title compound, with a view approximately along the b axis. N—H···N hydrogen bonds are shown as dashed lines, and H atoms not involved in these interactions have been omitted. For clarity, only one of the disordered components of the AgI2 anions is shown.

**Fig. 3.**
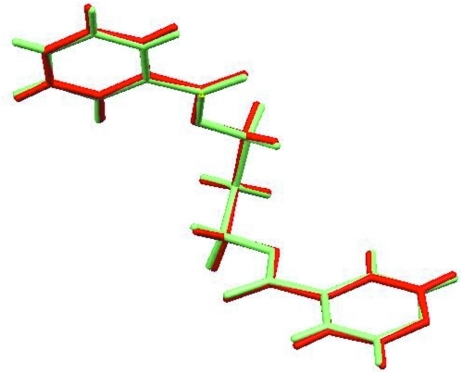
Superimposed structures for (I) and (II) (color code: green: (I); red: (II))

## supplementary crystallographic information

### Comment

The design of polymeric organic-inorganic materials with novel topologies and structural motifs is of current interest in the field of coordination chemistry (Blake *et al.*, 1999). This paper forms part of our continuing study of the synthesis, structural characterization and physical properties of coordination polymers (Brito *et al.*, 2011, Albanez *et al.*, 2011). The title compound, (I), was isolated during attempts to synthesize a coordination polymer by a self-assembly reaction between propane-1,3-diyl bis(pyridine-4-carboxylate) and AgI. The structure of the title compound, (I) consist of an organic 4-{[3-isonicotinoyloxy)propoxy]carbonyl}pyridinium cation which has a *gauche*-*gauche* (O/C/C/C—O/C/C/C or GG') conformation (Carlucci *et al.*, 2002) and lies on a twofold rotation axis, which passes through the central C atom of the aliphatic chain, and an inorganic (AgI_2_)^-^ anion, Fig, 1. In the anion, each silver atom is bound to two iodine atoms in a linear geometry. The anion was modelled assuming disorder around a crystallographic inversion centre near the location of the silver atom. The crystal packing is stabilized by a strong intermolecular N—H···N hydrogen bond, which links the cations into zigzag chains with graph-set notation C(16) (Bernstein *et al.*, 1995) running along the face diagonal of the *ac* plane (Fig. 2 and Table 1). There are only slight variations in the geometrical and conformational parameters between the cation complex of (I) and the unprotonated compound (Brito *et al.*, 2010), (II) so when both compounds are superimposed all related atoms fit within an RMSD of 0.0810 Å, Fig. 3 (Macrae *et al.*, 2008).

### Experimental

A solution of AgI (23.5 mg, 0.1 mmol) in water was slowly added to a solution of propane-1,3-diyl bis(pyridine-4-carboxylate) (28.6 mg, 0.1 mmol) in acetonitrile (4 ml), in presence of an excess of KI. Red single crystals suitable for X-ray analysis were obtained after a few days. Only a few single crystals were obtained due to low yield of the reaction, and no spectroscopic data were recorded.

### Refinement

H atoms were located in a difference map but finally geometrically positioned and refined using a riding model with fixed individual displacement parameters [*U*_iso_(H) = 1.2 *U*_eq_(C, N) and with C_aromatic_—H= 0.95 Å, N—H = 0.88 Å and C_methylene_—H = 0.99 Å]. The H atom bonded to N is disordered over two equally occupied sites. The (AgI_2_)^-^ anion was modelled assuming disorder around an inversion center. Reflections (1 1 2) and (0 0 4), were omitted due to their large disagreement between Fobs and Fcalc.

### Crystal data

**Table:**

(C_15_H_15_N_2_O_4_)[AgI_2_]	*F*(000) = 1216
*M**_r_* = 648.96	*D*_x_ = 2.171 Mg m^−^^3^
Monoclinic, *C*2/*c*	Mo *K*α radiation, λ = 0.71073 Å
Hall symbol: -C 2yc	Cell parameters from 9489 reflections
*a* = 14.8788 (7) Å	θ = 2.8–27.5°
*b* = 5.4712 (3) Å	µ = 4.14 mm^−^^1^
*c* = 24.5008 (11) Å	*T* = 173 K
β = 95.347 (4)°	Plate, red
*V* = 1985.81 (17) Å^3^	0.22 × 0.13 × 0.10 mm
*Z* = 4	

### Data collection

**Table:**

Stoe IPDS II two-circle diffractometer	2158 independent reflections
Radiation source: fine-focus sealed tube	1821 reflections with *I* > 2σ(*I*)
graphite	*R*_int_ = 0.053
ω scans	θ_max_ = 27.1°, θ_min_ = 2.8°
Absorption correction: multi-scan (*MULABS*; Spek, 2009; Blessing, 1995)	*h* = −18→18
*T*_min_ = 0.463, *T*_max_ = 0.682	*k* = −6→6
9000 measured reflections	*l* = −27→31

### Refinement

**Table:**

Refinement on *F*^2^	Primary atom site location: structure-invariant direct methods
Least-squares matrix: full	Secondary atom site location: difference Fourier map
*R*[*F*^2^ > 2σ(*F*^2^)] = 0.041	Hydrogen site location: inferred from neighbouring sites
*wR*(*F*^2^) = 0.119	H-atom parameters constrained
*S* = 1.03	*w* = 1/[σ^2^(*F*_o_^2^) + (0.0748*P*)^2^ + 3.0902*P*] where *P* = (*F*_o_^2^ + 2*F*_c_^2^)/3
2158 reflections	(Δ/σ)_max_ < 0.001
124 parameters	Δρ_max_ = 1.23 e Å^−^^3^
0 restraints	Δρ_min_ = −0.76 e Å^−^^3^

### Special details

**Table:**

Geometry. All e.s.d.'s (except the e.s.d. in the dihedral angle between two l.s. planes) are estimated using the full covariance matrix. The cell e.s.d.'s are taken into account individually in the estimation of e.s.d.'s in distances, angles and torsion angles; correlations between e.s.d.'s in cell parameters are only used when they are defined by crystal symmetry. An approximate (isotropic) treatment of cell e.s.d.'s is used for estimating e.s.d.'s involving l.s. planes.
Refinement. Refinement of *F*^2^ against ALL reflections. The weighted *R*-factor *wR* and goodness of fit *S* are based on *F*^2^, conventional *R*-factors *R* are based on *F*, with *F* set to zero for negative *F*^2^. The threshold expression of *F*^2^ > σ(*F*^2^) is used only for calculating *R*-factors(gt) *etc*. and is not relevant to the choice of reflections for refinement. *R*-factors based on *F*^2^ are statistically about twice as large as those based on *F*, and *R*- factors based on ALL data will be even larger.

### Fractional atomic coordinates and isotropic or equivalent isotropic displacement parameters (Å^2^)

**Table:**

	*x*	*y*	*z*	*U*_iso_*/*U*_eq_	Occ. (<1)
Ag1	0.0035 (2)	0.5344 (4)	0.50664 (10)	0.0480 (4)	0.50
I1	0.0644 (2)	0.7876 (5)	0.60644 (12)	0.0697 (6)	0.50
I2	−0.0570 (2)	0.2745 (6)	0.40629 (12)	0.0707 (6)	0.50
O1	0.10823 (19)	0.3522 (6)	0.79400 (11)	0.0484 (6)	
O2	0.1809 (2)	0.0578 (5)	0.75220 (11)	0.0488 (7)	
C1	0.1569 (2)	0.1523 (7)	0.79293 (15)	0.0378 (7)	
C2	0.0819 (3)	0.4755 (8)	0.74215 (17)	0.0467 (9)	
H2A	0.0679	0.3539	0.7126	0.056*	
H2B	0.1315	0.5813	0.7318	0.056*	
C3	0.0000	0.6262 (11)	0.7500	0.0485 (12)	
H3A	−0.0133	0.7329	0.7176	0.058*	
C11	0.1794 (2)	0.0487 (7)	0.84926 (15)	0.0389 (7)	
C12	0.2319 (3)	−0.1588 (8)	0.85514 (16)	0.0466 (8)	
H12	0.2521	−0.2369	0.8239	0.056*	
C13	0.2549 (4)	−0.2519 (8)	0.90675 (19)	0.0539 (10)	
H13	0.2926	−0.3921	0.9110	0.065*	
N14	0.2250 (3)	−0.1481 (8)	0.95113 (14)	0.0548 (9)	
H14	0.2395	−0.2113	0.9838	0.066*	0.50
C15	0.1735 (3)	0.0493 (9)	0.94599 (18)	0.0554 (10)	
H15	0.1531	0.1200	0.9780	0.066*	
C16	0.1487 (3)	0.1559 (9)	0.89617 (17)	0.0496 (9)	
H16	0.1118	0.2980	0.8935	0.060*	

### Atomic displacement parameters (Å^2^)

**Table:**

	*U*^11^	*U*^22^	*U*^33^	*U*^12^	*U*^13^	*U*^23^
Ag1	0.0385 (5)	0.0570 (12)	0.0501 (11)	0.0024 (7)	0.0122 (7)	0.0237 (7)
I1	0.0575 (7)	0.0881 (15)	0.0641 (12)	0.0079 (8)	0.0091 (7)	0.0217 (8)
I2	0.0617 (7)	0.0853 (14)	0.0660 (13)	−0.0011 (7)	0.0104 (8)	0.0195 (8)
O1	0.0526 (15)	0.0558 (16)	0.0368 (13)	0.0087 (13)	0.0033 (11)	−0.0008 (12)
O2	0.0654 (18)	0.0475 (15)	0.0346 (13)	0.0040 (13)	0.0098 (12)	0.0017 (12)
C1	0.0356 (16)	0.0426 (18)	0.0354 (17)	−0.0036 (14)	0.0042 (13)	−0.0003 (15)
C2	0.043 (2)	0.052 (2)	0.045 (2)	0.0030 (16)	0.0026 (16)	0.0053 (17)
C3	0.041 (3)	0.048 (3)	0.056 (3)	0.000	0.003 (2)	0.000
C11	0.0388 (17)	0.0441 (18)	0.0345 (17)	−0.0064 (14)	0.0069 (13)	−0.0018 (15)
C12	0.056 (2)	0.046 (2)	0.0383 (19)	−0.0009 (17)	0.0077 (16)	−0.0010 (16)
C13	0.071 (3)	0.047 (2)	0.043 (2)	0.0023 (18)	0.0070 (19)	0.0031 (17)
N14	0.067 (2)	0.061 (2)	0.0355 (17)	−0.0051 (19)	0.0039 (15)	0.0048 (16)
C15	0.060 (2)	0.072 (3)	0.0348 (19)	−0.003 (2)	0.0105 (17)	−0.006 (2)
C16	0.051 (2)	0.061 (2)	0.0378 (19)	0.0045 (19)	0.0076 (15)	−0.0029 (18)

### Geometric parameters (Å, °)

**Table:**

Ag1—I1	2.882 (3)	C11—C12	1.378 (6)
Ag1—I2	2.909 (3)	C11—C16	1.404 (5)
O1—C1	1.313 (5)	C12—C13	1.376 (6)
O1—C2	1.459 (5)	C12—H12	0.9500
O2—C1	1.207 (5)	C13—N14	1.339 (6)
C1—C11	1.501 (5)	C13—H13	0.9500
C2—C3	1.498 (5)	N14—C15	1.322 (6)
C2—H2A	0.9900	N14—H14	0.8800
C2—H2B	0.9900	C15—C16	1.372 (6)
C3—C2^i^	1.498 (5)	C15—H15	0.9500
C3—H3A	0.9900	C16—H16	0.9500
			
I1—Ag1—I2	179.44 (19)	C16—C11—C1	122.2 (4)
C1—O1—C2	118.1 (3)	C13—C12—C11	119.4 (4)
O2—C1—O1	125.4 (3)	C13—C12—H12	120.3
O2—C1—C11	122.8 (3)	C11—C12—H12	120.3
O1—C1—C11	111.9 (3)	N14—C13—C12	121.1 (4)
O1—C2—C3	107.4 (3)	N14—C13—H13	119.5
O1—C2—H2A	110.2	C12—C13—H13	119.5
C3—C2—H2A	110.2	C15—N14—C13	120.2 (4)
O1—C2—H2B	110.2	C15—N14—H14	119.9
C3—C2—H2B	110.2	C13—N14—H14	119.9
H2A—C2—H2B	108.5	N14—C15—C16	122.5 (4)
C2—C3—C2^i^	113.2 (5)	N14—C15—H15	118.8
C2—C3—H3A	109.0	C16—C15—H15	118.8
C2^i^—C3—H3A	108.8	C15—C16—C11	118.0 (4)
C12—C11—C16	118.9 (4)	C15—C16—H16	121.0
C12—C11—C1	118.9 (3)	C11—C16—H16	121.0
			
C2—O1—C1—O2	−1.3 (6)	C16—C11—C12—C13	−1.7 (6)
C2—O1—C1—C11	179.4 (3)	C1—C11—C12—C13	178.3 (4)
C1—O1—C2—C3	157.3 (4)	C11—C12—C13—N14	1.9 (7)
O1—C2—C3—C2^i^	−68.0 (3)	C12—C13—N14—C15	−1.0 (7)
O2—C1—C11—C12	2.1 (6)	C13—N14—C15—C16	0.0 (7)
O1—C1—C11—C12	−178.6 (3)	N14—C15—C16—C11	0.2 (7)
O2—C1—C11—C16	−177.9 (4)	C12—C11—C16—C15	0.7 (6)
O1—C1—C11—C16	1.4 (5)	C1—C11—C16—C15	−179.3 (4)

Symmetry codes: (i) −*x*, *y*, −*z*+3/2.

### Hydrogen-bond geometry (Å, °)

**Table:**

*D*—H···*A*	*D*—H	H···*A*	*D*···*A*	*D*—H···*A*
N14—H14···N14^ii^	0.88	1.80	2.684 (7)	176.

Symmetry codes: (ii) −*x*+1/2, −*y*−1/2, −*z*+2.
